# Dried Apricot Polyphenols Suppress the Growth of A549 Human Lung Adenocarcinoma Cells by Inducing Apoptosis via a Mitochondrial-Dependent Pathway

**DOI:** 10.3390/foods14010108

**Published:** 2025-01-02

**Authors:** Caiyun Zhao, Jingteng Wang, Jintian Guo, Wenjing Gao, Bin Li, Xin Shang, Li Zheng, Bin Wu, Yinghua Fu

**Affiliations:** 1 Xinjiang Key Laboratory of Biological Resources and Genetic Engineering, College of Life Science and Technology, Xinjiang University, Urumqi 830017, China; zcy13565816792@163.com (C.Z.); 18769520685@163.com (J.W.); guojintian99@163.com (J.G.); gaowj0923@163.com (W.G.); 18242180424@163.com (B.L.); sshangxin0919@163.com (X.S.); xjdxzl01@163.com (L.Z.); 2 Institute of Agro-Products Storage and Processing, Xinjiang Academy of Agricultural Sciences, Urumqi 830091, China

**Keywords:** dried apricot polyphenols, A549, inhibition, apoptosis

## Abstract

Dried apricots are rich in a variety of polyphenols, which have anti-cancer activity. In this study, 949 phenolic substances were found by means of UPLC-MS/MS, mainly including 2′,7-dihydroxy-3′,4′-dimethoxyisoflavan, scopoletin, rutin, quercetin-3-O-robinobioside, and elaidolinolenic acid. The results indicated that dried apricot polyphenols (DAPs) could cause cell cycle arrest in the G0/G1 and G2/M phases by decreasing the cyclin D1, CDK4, cyclin B1, CDK1, and CDK6 levels in A549 human lung adenocarcinoma cells. Moreover, the ROS and Bax levels were increased, and the Bcl-2 and mitochondrial membrane potential were decreased in A549 cells treated with DAP, increasing caspase-9, caspase-3, and cleaved-PARP1 activities and leading to apoptosis of the A549 cells. Meanwhile, tumor growth was also inhibited by DAPs in an A549 tumor-bearing mouse model, Bax and caspase-3 were upregulated, and Bcl-2 was downregulated, inducing apoptosis of lung cancer cells. In conclusion, DAPs could inhibit lung cancer cell growth by inducing apoptosis due to cell cycle arrest and mitochondria-dependent pathways.

## 1. Introduction

Lung cancer has emerged as a predominant malignancy in the 21st century, posing a significant hazard to human health. In 2023, around 1.8 million deaths were ascribed to lung cancer, representing 18% of all cancer-related deaths [1]. Currently, the principal strategies for cancer treatment are radiation, chemotherapy, surgery, immunotherapy, and targeted therapeutics [2,3]. Nonetheless, a major issue associated with the extended use of chemotherapeutic agents is the emergence of harmful side effects and drug resistance [4]. Since the 1950s, plant-derived natural products have attracted significant interest as valuable sources of anti-tumor agents due to their low toxicity and limited side effects. Chief among these are polyphenolic compounds such as flavonoids, terpenoids, and alkaloids, recognized for their potential antioxidant properties and capacity to induce apoptosis in tumor cells. By meticulously analyzing data spanning several decades, Cragg et al. summarized the substantial contributions of natural products and their derivatives to the development of new pharmaceuticals, emphasizing the necessity of continued exploration and utilization of these resources [5]. Studies have found that polyphenols produce pleiotropic effects by influencing many cellular signaling pathways that suppress lung cancer growth. Carvacrol nanoemulsions could induce A549 cells to produce reactive oxygen species (ROS), activating key regulators of apoptosis and caspase cascades and displaying strong anti-tumor potential in a nude mouse model [6]. Ellagic acid has been shown to inhibit lung cancer cell proliferation in vitro and in vivo, reducing mitochondrial intima potential and oxygen consumption [7]. Additionally, *Phellinus baumii* polyphenols were found to exert anti-tumor effects by triggering apoptosis, producing cell cycle arrest, accumulating ROS, and decreasing the mitochondrial membrane potential (MMP) in A549 cells [8].

Apricot (*Prunus armeniaca* L.), commonly known as sweet plum, belongs to the Rosaceae family [9]. Apricot contains an abundance of bioactive phenolic substances such as non-eaten menic acid, chlorogenic acid, neo-chlorogenic acid, quercetin, and rutin. Chlorogenic acid, rutin, (+)-catechin, neochlorogenic acids, (−)-epicatechin, quercetin-3-glycosides, and procyanidins have been identified as the main phenolic compounds in nine apricot cultivars [10]. Thirteen individual phenolic compounds were detected in four apricot cultivars in Latvia, with catechin, chlorogenic acid, rutin, epicatechin, and neochlorogenic acid as the most abundant [11]. Polyphenolic compounds from 14 apricot types in Northern India were examined with high-performance liquid chromatography-mass spectrometry, identifying catechin, epicatechin, quercetin, kaempferol, naringin, apigenin, luteolin, vitexin, isovitexin, and rutin [12]. Moreover, Hattori et al. found that apricot extracts exhibited an anti-tumor efficacy both in vitro and in vivo via a ROS-dependent mechanism [13]. Yamai et al. showed that triterpenes in apricots enhanced the inhibitory and anti-metastatic effect of anti-cancer drugs on human esophageal cancer cells [14]. Mori et al. demonstrated that Japanese apricot induced strong autophagy in colon cancer cells in vitro [15]. Nakagawa et al. determined that apricots effectively repressed the proliferation of breast cancer cells in vitro, potentially via cell cycle modification and the activation of apoptosis [16]. Apricots have been demonstrated to effectively inhibit the proliferation of non-small cell lung cancer (NSCLC) cells [17].

In this study, the effects of dried apricot polyphenols (DAPs) on A549 cell vitality, cell cycles, apoptosis, cell-activated oxygen, mitochondrial film potential, and apoptosis-related protein expression were investigated through in vitro experiments to explore their anti-lung cancer mechanism. At the same time, the impacts of DAPs on tumor growth in tumor-bearing mice as well as the expression levels of Ki67, Bax, Bcl-2, and caspase-3 proteins were investigated to clarify the inhibitory effect on A549 cells.

## 2. Materials and Methods

### 2.1. Materials

Saimaiti dried apricots were purchased from Yingjisha County, Kashgar, Xinjiang in China. Cisplatin was supplied by Yuanye Bio-Technology Co., Ltd. (Shanghai, China). Solarbio Technology Co., Ltd. (Beijing, China) provided the CCK-8 assay kits. The remaining reagents were all commercially accessible and of analytical grade. Cell Bioscience Inc. (Shanghai, China) supplied the A549 human lung adenocarcinoma cells, which were cultured in DMEM media with 10% fetal bovine serum and 1% penicillin-streptomycin in an incubator set at 37 °C with 5% CO_2_.

### 2.2. Preparation of Dried Apricot Polyphenols

After the apricots were cored, they were laid flat on oven screens and dried with hot air at 50 °C. The dried apricots were then ground into a powder using a grinder. A 70% ethanol solution was mixed with the dried apricot powder at a rate of 1:21 (g/mL) solids to liquids. After 20 min of ultrasonic treatment at 200 W, the mixture was extracted for 2.6 h at 51 °C in a water bath. The ethanol extraction was centrifuged at 8000 rpm for 20 min, and the supernatant was collected and concentrated with a rotavapor device. Then, the concentrated liquid was enriched with AB-8 macroporous resin, and the eluate was gathered and further concentrated. Finally, the dried apricot polyphenols (DAPs) were created by freeze-drying them in a vacuum.

### 2.3. Analysis of Dried Apricot Polyphenols via UPLC-MS/MS

Ultra-performance liquid chromatography-tandem mass spectrometry (UPLC-MS/MS) was used to examine the polyphenolic compounds in the dried apricot polyphenols (DAPs). A total of 50 mg of DAPs was dissolved in 1200 µL of 70% methanol. The supernatants were filtered after centrifugation and used for UPLC-MS/MS analysis. The mass spectrometry parameters for the 30 polyphenols are provided in Table S1.

### 2.4. CCK-8 Assay

We collected logarithmic growth phase A549 cells and adjusted the cell density to about 1 × 10^5^ cells/mL, then seeded 100 µL of the cell suspension into each well of a 96-well plate and cultured it for 24 h. Following that, the cells were exposed to varying concentrations of DAPs (200, 400, 600, 800, 1000, and 1200 µg/mL), with cisplatin (20 µg/mL) serving as the positive control, and six holes were created in each group. After 24, 48, and 72 h, CCK-8 solution was added to each well, and the reaction was shielded from light for 30 min before the absorbance at 450 nm was determined.

### 2.5. Wound Healing Assay

The A549 cells were harvested and evenly seeded into a 12-well plate. Upon achieving nearly 100% confluence in the cell monolayers, a scratch was made on the cell surface. Various DAP concentrations were administered to each well for 24 h treatment of the cells.

The migration extent of the A549 cells at 0 and 24 h was imaged using a microscope, and the scratch area of the samples was evaluated using ImageJ software (https://imagej.net/ij/, accessed on 22 November 2024). The formula for estimating wound healing percentages is:

(1) Wound healing (%)=(1−scratch area at 24 h/scratch area at 0 h)×100%

### 2.6. Analysis of Cell Cycle and Apoptosis

In 6-well plates, 1.5 × 10^5^ A549 cells were placed into each well and exposed to several doses (600, 800, 1000, and 1200 µg/mL) of DAPs over 24 h. After collection, the cells were resuspended in 70% cold ethanol and fixed at 4 °C for 30 min. The A549 cells were then stained with PI stain and cultured at 37 °C in the dark for 30 min to study the cell cycle. We used the Annexin V-FITC/propidium iodide (PI) Apoptosis Detection Kit according to the manufacturer’s instructions to examine apoptosis in the A549 cells. The use of flow cytometry allowed for the examination of cell cycle dispersion and apoptosis.

### 2.7. Hoechst 33258 Staining

In 6-well plates, 1.5 × 10^5^ A549 cells were placed into each well and subjected to a 24 h DAP treatment at several doses (600, 800, 1000, and 1200 µg/mL). After that, 4% paraformaldehyde was used to fix the cells for 10 min; the fixative was then washed off with PBS. To examine the nuclear morphology of the cells with an inverted fluorescent microscope, Hoechst 33258 staining solution (1 μg/mL) was added.

### 2.8. Analysis of MMP and ROS

The A549 cells were inoculated into 6-well plates at a density of 1.5 × 10^5^ cells per well and given different amounts (600, 800, 1000, and 1200 µg/mL) of DAPs for 24 h. Subsequently, the cells were collected and stained with the JC-1 fluorescence probe, then incubated for 20 min at 37 °C. Under inverted fluorescent microscopy, the MMP of the cells was noted. In addition, the number of ROS in the A549 cells was determined by examining the 20,70-dichlorofluorescein diacetate (DCFH-DA) fluorescence intensity. The A549 cells were harvested in serum-free media and incubated with DCFH-DA for 20 min at 37 °C. In accordance with the kit instructions, flow cytometry and inverted fluorescence microscopy were used to assess and evaluate the ROS levels in the A549 cells.

### 2.9. Enzyme-Linked Immunosorbent Assays

Proteins were recovered from the A549 cells after treatment with varying doses of DAPs. The protein concentration was measured using the BCA method. The levels of CDK1, CDK4, CDK6, cyclin B, and cyclin D1 as well as Bax, Bcl-2, caspase-9, caspase-3, PARP, and PARP1 were quantified utilizing ELISA kits (Jianglai Biology Co., Ltd., Shanghai, China).

### 2.10. In Vivo Animal Study

The Animal Ethics Committee of Xinjiang University approved all of the tests on animals in this work (Approval No.: XJUAE-2023-021). The experimental animals used in this study were male BALB/c nude mice weighing about 18 g, which were provided by Nanjing Junke Biotechnology Co., Ltd., Nanjing, China (Certificate No.: B202312040363).

**Figure 1:**
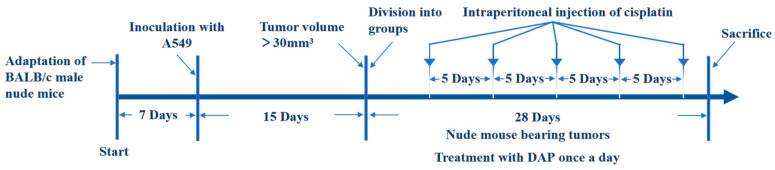
Flowchart of the animal experiment design.

All mice were kept under controlled temperatures (25 ± 2 °C) and relative humidity (50–70%) on a 12/12 light/dark cycle with free access to water and a normal diet. After a week of adjustment to the environment, 10 mice were randomly selected as the normal group (NC), and all others were inoculated with 100 μL of A549 cell suspension (1 × 10^6^ cell/mouse) in the right axilla to create a mouse model with an A549 tumor. The volume of the tumor was calculated as AB^2^/2, where A is the tumor’s largest width and B is its smallest. After 15 d, the mice that were successfully modeled were randomly split into four groups (*n* = 5). These groups were the model group (MC), the dried apricot polyphenol low-dose group (DAP-L), the dried apricot polyphenol high-dose group (DAP-H), and the positive control group (PC). The NC and MC groups were gavaged with 0.1 g/mL saline (0.9%). Mice in the DAP-L and DAP-H groups received 400 and 600 mg/kg/day of DAPs orally in gavage, respectively. The PC group was intraperitoneally injected with cisplatin (5 mg/kg/5 day). The weight and tumor volume of the mice were measured during the experiment. The whole experiment lasted 28 d. Blood samples were collected from the mouse eyeballs for serum preparation and biochemical analysis. Then, the mice were sacrificed via cervical dislocation, and the heart, liver, spleen, lung, kidney, and tumor tissues of the mice were taken immediately and stored in formaldehyde solution for subsequent experiments. Figure 1 shows the flowchart of the animal experiment design.

### 2.11. Analysis of Serum Biochemicals

After the 28 d experiment, serum was collected from the mice, and corresponding assay kits were used to quantify the blood urea nitrogen (BUN), creatinine (CR), aspartate aminotransferase (AST), and alanine aminotransferase (ALT) levels.

### 2.12. Immunohistochemical Analysis of Ki67 Protein

The paraffin-embedded blocks of tumor tissues were deparaffinized, the antigenic repair was performed with citric acid repair buffer, and the sections were sealed. Then, the tissue sections were blocked using the main antibody anti-Ki67 and left overnight at 4 °C. The slices were developed with DAB colorant, incubated with a biotin-labeled secondary antibody at 37 °C, and counterstained with hematoxylin. After mounting under a microscope, the sections were examined to evaluate the Ki67 protein expression in several groups.

### 2.13. Determination of Protein Content in Tumor Tissue

The tumor tissues of the lung cancer mice were snap-frozen in liquid nitrogen, a specific volume of PBS and protease inhibitors were added, and then homogenized using a homogenizer. The homogenate was sonicated to create a uniform slurry and centrifuged for 20 min at 15,000× *g*. The supernatant was carefully gathered and examined for the presence of the proteins Bax, Bcl-2, and caspase-3 with ELISA kits.

### 2.14. Statistical Analysis

Every experiment conducted in this study had a minimum of three independent replicates. GraphPad Prism version 8.0.2 was used to analyze all experimental data, which were shown as the mean ± standard deviation (mean ± SD). A one-way analysis of variance (ANOVA) with Duncan’s multiple range test was used to compare the differences among various groups. Data differences were deemed significant at the statistical significance threshold of *p* < 0.05.

## 3. Results and Discussion

### 3.1. Compositions of Dried Apricot Polyphenols

In the study, dried apricot polyphenols (DAPs) were enriched using AB-8 macroporous resin, and the UPLC-MS/MS analysis determined that flavonoids, phenolic acids, lignans, coumarins, stilbenes, and tannins were the major phenolic substances, accounting for 48.86%, 28.39%, 12.52%, 5.11%, 5.04%, and 0.08%, respectively. A total of 949 polyphenolic compounds were identified including 2′,7-dihydroxy-3′,4′-dimethoxyisoflavan, scopoletin, rutin, quercetin-3-O-robinobioside, and elaidolinolenic acid (Figure S1 and Table S2). In addition, the dried apricot polyphenols contained caffeic acid, gallic acid, neochlorogenic acid, ferulic acid, quercetin, cinnamic acid, and chlorogenic acid. Myricetin, rutin, *p*-coumaric acid, chlorogenic acid, and gallic acid were reported to be the main phenolic compounds in dried apricots from Malatya, Turkey [18]. Muttalip Gundogdu et al. identified epigallocatechin, chlorogenic acid, rutin, pyrogallol, caffeic acid, and catechol among the eight apricot cultivars grown in the Malatya region of Turkey [19]. Using high-performance liquid chromatography, Vega-Gálvez et al. identified the phenolic compounds in Chilean apricots as including quercetin 3-rutinoside, catechin, chlorogenic acid, epicatechin, and syringic acid [20]. Moreover, these polyphenol components were confirmed by domestic and international studies as bioactive substances with the potential for anti-lung cancer activity. The polyphenol scopolide, isolated from the alcohol extract from the stem bark of the tree orchid, has been shown to inhibit A549 cells, inducing early and late apoptosis [21]. Additionally, quercetin has been reported to promote apoptosis mediated by Bcl-2/Bax as well as mitotic arrest, which consequently inhibited the growth and migration of A549 cells [22].

### 3.2. DAPs Decreased the Viability of A549 Cells

**Figure 2:**
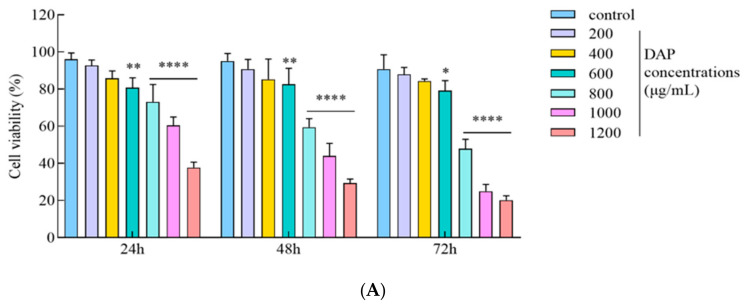

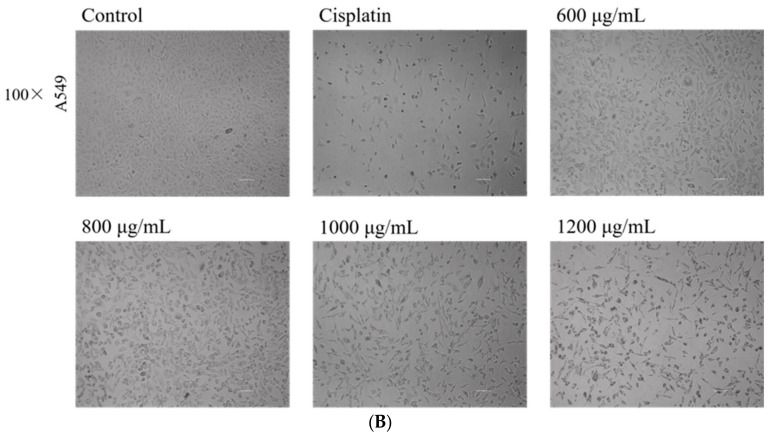
Effect of DAPs on A549 cell activity. (**A**) Cell viability assessed with CCK-8 assay; (**B**) cell morphology observed under inverted microscope, scale bar: 100μm. * *p* < 0.05, ** *p* < 0.01, **** *p* < 0.0001 compared to the control group.

The A549 cells were subjected to varying DAP concentrations for 24, 48, and 72 h. The anti-tumor effects of the DAPs were evaluated using the CCK-8 test to gauge cell viability. Figure 2A illustrates that the viability of the A549 cells exposed to DAP doses ranging from 600 to 1200 μg/mL was markedly diminished (*p* < 0.01) in a dose- and time-dependent manner relative to the control group. Additionally, morphological examination of the A549 cells using an inverted microscope (Figure 2B) indicated that the DAP-treated A549 cells exhibited a shrunken and rounded morphology, with a decreased cell count relative to the control group. Hattori et al. found that Japanese apricot extracts demonstrated a concentration-dependent inhibitory impact on A549 cells [13], indicating that dried apricot polyphenols could be valuable as an additional treatment for cancer in humans.

### 3.3. DAPs Suppressed the Migration of A549 Cells

**Figure 3:**
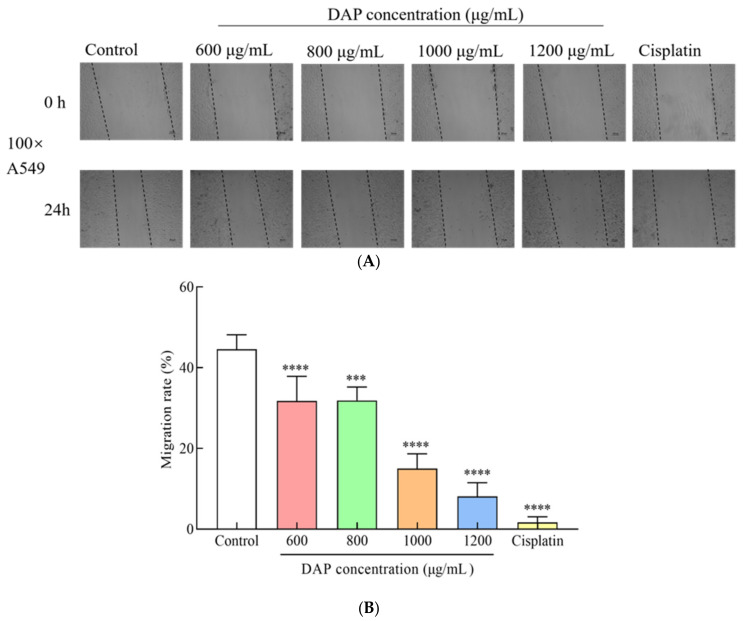
Effect of DAPs on A549 cell migration. (**A**) Migration of A549 cells observed with inverted microscope, scale bar: 100 μm; (**B**) analysis of A549 cell migration using Image J. *** *p* < 0.001, **** *p* < 0.0001 compared to the control group.

The migration of cancer cells is a pivotal element in metastasis, which contributes to cancer-related mortality. This study used the wound healing test to evaluate the impact of DAPs on A549 cell migration. Figure 3 illustrates that DAPs at concentrations of 600, 800, 1000, and 1200 μg/mL markedly suppressed the migration of A549 cells (*p* < 0.001) relative to the control group, with migration rates reduced by 12.85%, 12.71%, 29.57%, and 36.44%, respectively. These findings suggest that DAPs could impede the migration of A549 cells, demonstrating their potential for anti-migratory activity. The leaves of white mulberry were full of bioactive phenolic substances that were shown to prevent cell migration and invasion, in addition to having notable cytotoxic effects on A549 cells [23]. In addition, gossypol, as a natural polyphenolic compound, dramatically reduced H1299 and A549 cell survival, motility, and invasion [24].

### 3.4. DAPs Induced Apoptosis of A549 Cells

**Figure 4:**
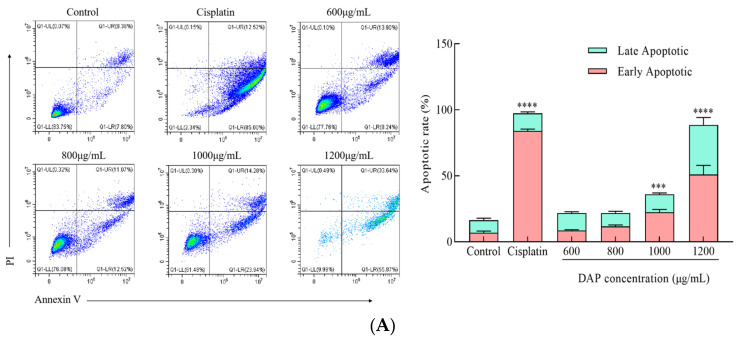

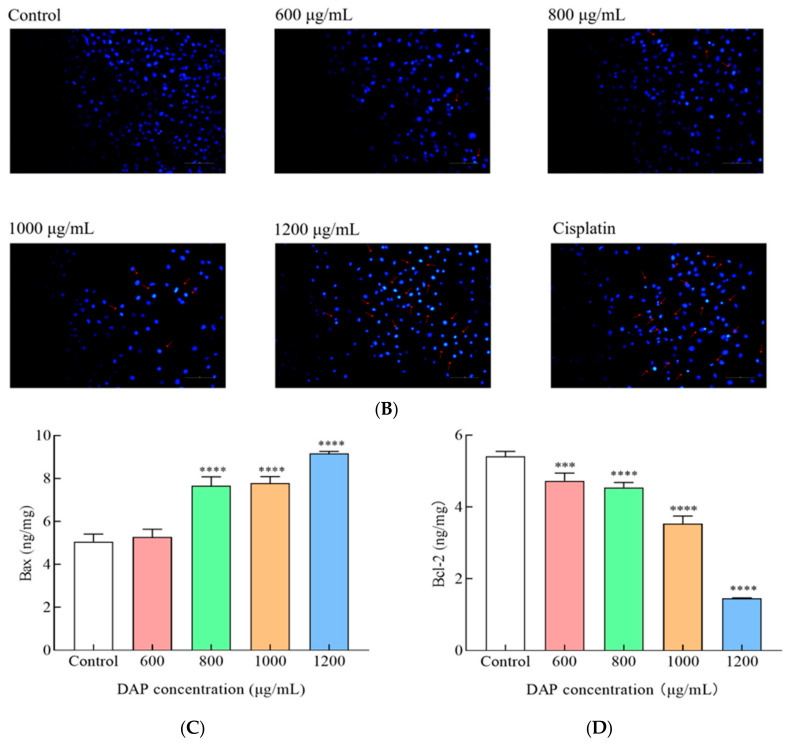
Effect of DAPs on apoptosis in A549 cells. (**A**) Apoptosis rate analyzed via flow cytometry; (**B**) nuclear morphology observed under fluorescence microscope, scale bar: 100 μm; (**C**) Bax; (**D**) Bcl-2. *** *p* < 0.001, **** *p* < 0.0001 compared to the control group.

As seen in Figure 4A, flow cytometry indicated a significant increase (*p* < 0.001) in A549 cell apoptosis at DAP concentrations of 1000 and 1200 μg/mL compared to the control group. Under the inverted fluorescent microscope, the control A549 cell nuclei were spherical, undamaged, and evenly stained (Figure 4B). However, when the DAP concentration increased, the A549 cells were reduced and their nuclear structure changed including chromatin condensation and disintegration. Apoptosis was also linked to B-cell lymphoma-2 (BCL-2) protein family expression. As shown in Figure 4C,D, DAPs significantly upregulated Bax (*p* < 0.0001) and downregulated Bcl-2 (*p* < 0.001) in the A549 cells compared to the control. These findings suggest that DAPs elevated the Bax and Bcl-2 protein levels and caused apoptosis, which killed the A549 cells. By lowering the Bcl-2/Bax ratio, the ethanol extract of the plant Medicago orbicularis was shown to promote cell death [25]. Çebi et al. reported that Turkish hazelnut leaf extracts were rich in polyphenols, inhibited lung cancer cell migration, and caused apoptosis in cells treated with a methanolic extract [26].

### 3.5. DAPs Induced Cell Cycle Arrest in A549 Cells

**Figure 5:**
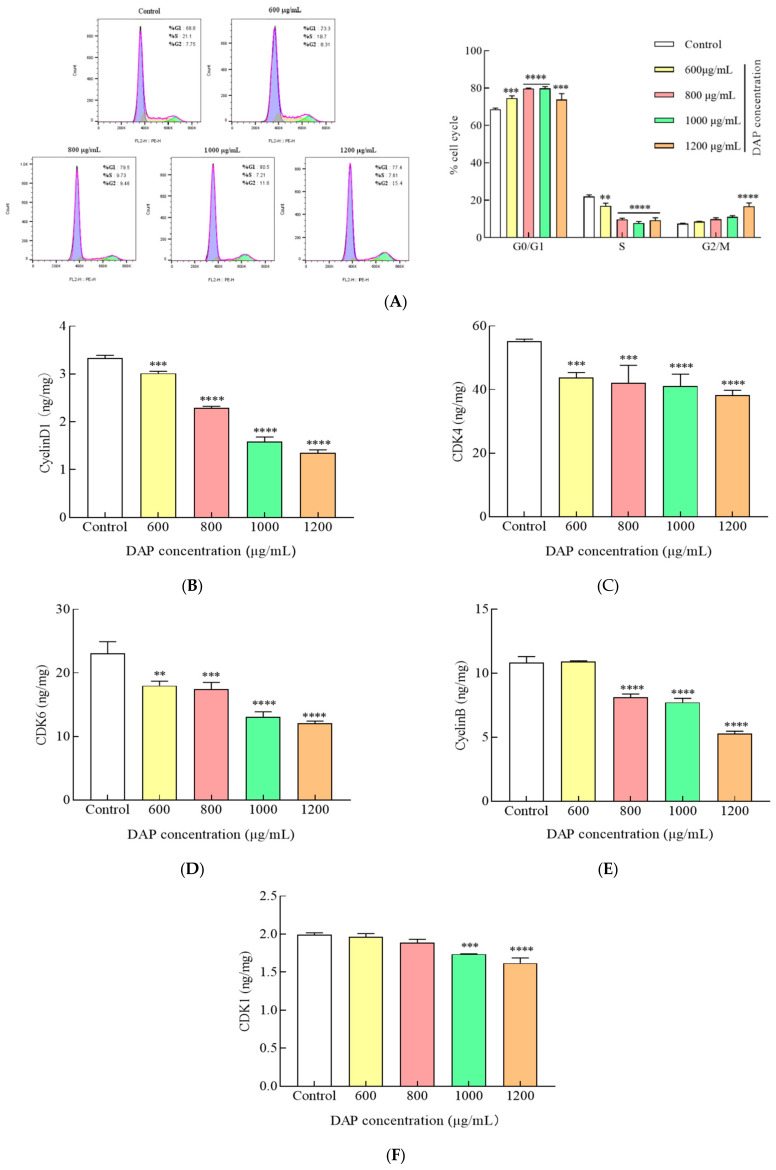
Effect of DAPs on the A549 cell cycle. (**A**) The cell cycle distribution was detected via flow cytometry; (**B**) cyclin D1; (**C**) CDK4; (**D**) CDK6; (**E**) cyclin B; (**F**) CDK1. ** *p* < 0.01, *** *p* < 0.001, **** *p* < 0.0001 compared to the control group.

The impact of DAPs on the A549 cell cycle distribution was analyzed using flow cytometry. Figure 5A illustrates that the proportion of A549 cells in the G0/G1 phase increased substantially from 68.67% to 79.85% after treatment with 1000 μg/mL DAPs (*p* < 0.0001), and the proportion of cells in the G2/M phase rose considerably from 7.42% to 16.82% after treatment with 1200 μg/mL DAPs (*p* < 0.0001). Cyclins and cyclin-dependent kinases (CDKs) regulate the cell cycle through a complicated protein network. The DAP treatment significantly reduced the cyclin D1, CDK4, and CDK6 expression levels in the A549 cells (*p* < 0.01) (Figure 5B–D), promoting the G1 phase transition to the S phase. Additionally, the expression levels of cyclin B and CDK1, linked to the G2/M phase, were also significantly reduced (*p* < 0.01) (Figure 5E,F). DAPs downregulated the synthesis of cyclin D1, CDK4, CDK6, cyclin B, and CDK1, which arrested the cell cycle during the G0/G1 and G2/M phases in the A549 cells and might have caused cell death. Moreover, Martinez et al. reported that an acai berry alcohol extract might raise the number of A549 cells in the G0/G1 phase, which led to a notable rise in apoptosis [27]. Lin et al. found that polyphenols such as licochalcone A and ursolic acid caused autophagy and S and G2/M cell cycle arrest in A549 cells [28]. Additionally, Zhang et al. discovered that luteolin downregulated cyclin D1 expression, which resulted in apoptosis and G1 phase cell cycle arrest [29].

### 3.6. DAPs Reduced the Mitochondrial Membrane Potential (MMP) in A549 Cells

**Figure 6:**
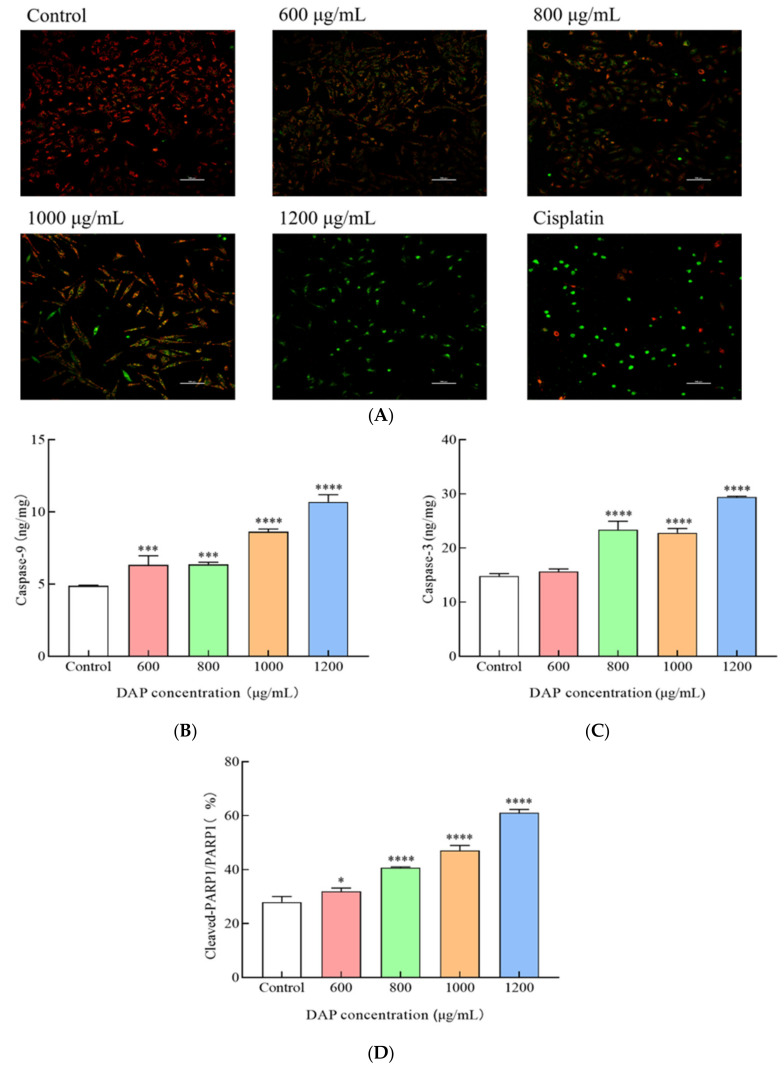
Effect of DAPs on the MMP of A549 cells. (**A**) The MMP observed under fluorescence microscope, scale bar: 100 μm; (**B**) caspase-9; (**C**) caspase-3; (**D**) cleaved-PARP1/PARP1. * *p* < 0.05, *** *p* < 0.001, **** *p* < 0.0001 compared to the control group.

The decline in the MMP is considered the earliest event in the apoptosis cascade and a hallmark of cell apoptosis [30]. The JC-1 probe was employed to examine the influence of DAPs on the MMP in A549 cells. JC-1 usually exists as a polymer in the normal mitochondrial matrix, producing red fluorescence. However, when MMP is reduced, JC-1 becomes a monomer and produces green fluorescence. In Figure 6A, under the inverted fluorescence microscope, the A549 cells in the control group exhibited red fluorescence, and those treated with DAPs showed a significant increase in green fluorescence intensity in a dose-dependent manner, indicating a reduction in the MMP. Subsequently, the stimulation level of the caspase cascade set off by the mitochondrial-dependent pathway was checked. As illustrated in Figure 6B–D, the ratio of cleaved-PARP1 to PARP1 protein rose dramatically (*p* < 0.05) in the A549 cells, while the caspase-3 and caspase-9 protein levels were dramatically boosted (*p* < 0.001) following the DAP treatment. This suggests that DAPs promoted PARP1 cleavage, leading to A549 cell apoptosis. These results indicate that DAPs might cause apoptosis in A549 cells through a route that depends on mitochondria. Moreover, Güçlü et al. found that by elevating caspase-3 and caspase-9 expression, tomentosin exhibited anti-cancer effects in pancreatic cancer cells [31].

### 3.7. DAPs Promoted Reactive Oxygen Species (ROS) Generation in A549 Cells

**Figure 7:**
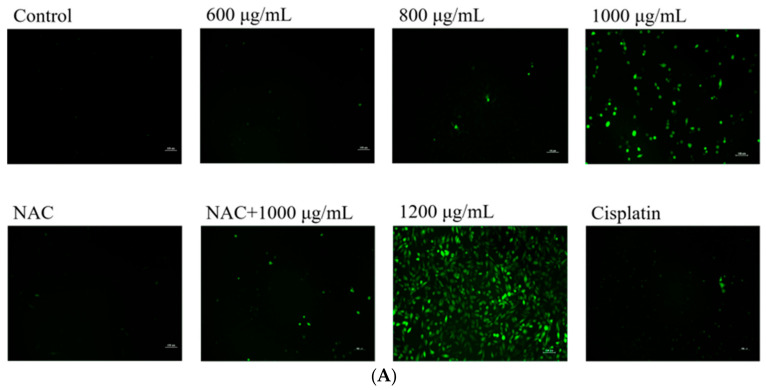

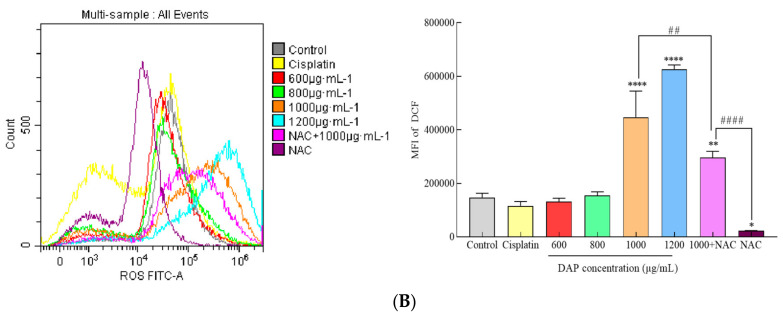
Effect of DAPs on ROS in A549 cells. (**A**) Fluorescence intensity of ROS assessed using fluorescence microscopy, scale bar: 100 μm; (**B**) the cellular ROS level quantified via flow cytometry. * *p* < 0.05, ** *p* < 0.01, **** *p* < 0.0001 compared to the control group; ## *p* < 0.01, #### *p* < 0.0001 compared to the 1000 μg/mL + NAC group.

The decline in the MMP results in elevated ROS levels within the cell. Excessive ROS disrupts the intracellular redox balance, and mitochondria are the primary sites of oxidative metabolism and oxidative stress. ROS are closely linked to mitochondrial-dependent apoptosis [32,33]. Under an inverted fluorescent microscope, a progressive increase in green fluorescence was seen as the DAP concentration increased (Figure 7A). Furthermore, flow cytometry was employed to identify the generation of ROS. Figure 7B illustrates that treatment with 1000 and 1200 μg/mL DAPs resulted in a considerable elevation in intracellular ROS levels (*p* < 0.0001). To validate that DAPs stimulate ROS formation in A549 cells, N-acetyl-L-cysteine (NAC), an inhibitor of ROS, was administered to the cells prior to the DAP treatment. The findings indicated that NAC markedly reduced the ROS levels induced by the DAPs in A549 cells (*p* < 0.01) and suggest that the generation of ROS might be connected to the inhibitory effect of DAPs on A549 cell activity. Moreover, the Japanese apricot alcohol extract was found to promote ROS accumulation in human pancreatic cancer cells, potentially offering therapeutic value in the treatment of cancer [13]. Similarly, Cheng et al. found that penduline, an isoflavone compound, could induce apoptosis and significantly reduce the viability of H1299 cells through ROS accumulation and mitochondrial dysfunction [34].

### 3.8. DAPs Suppressed Lung Cancer Cell Growth In Vivo

**Figure 8:**
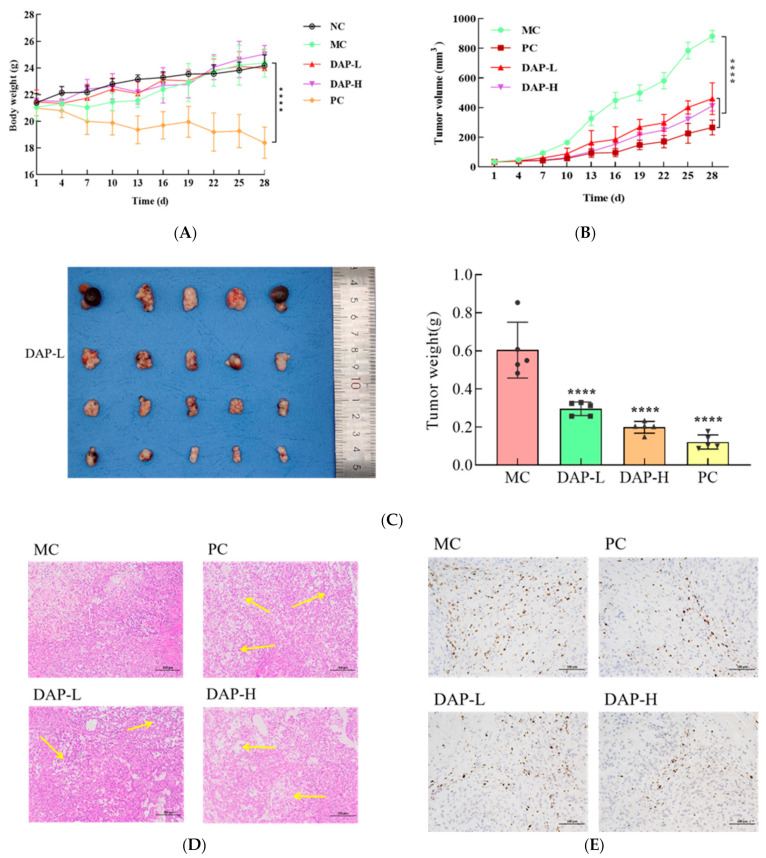
Anti-tumor effects of DAPs in vivo. (**A**) Variations in the body weight of mice during the experiment; (**B**) changes in tumor volume during the experiment; (**C**) tumor weight at the conclusion of the experiment; (**D**) pathological evaluation of the tumor tissue, scale bar: 200 μm; (**E**) Ki67 expression in the tumor tissue, scale bar: 100 μm. **** *p* < 0.0001 compared to the MC group.

A tumor-bearing mouse model was established using BALB/C nude mice to systematically assess the anti-tumor effect of DAPs on A549 cells. Fifteen days post injection of the A549 cells, the tumor-bearing mice underwent treatment with DAPs for 28 d. Throughout this period, both the body weight of the mice and the size of the tumors were systematically monitored at designated time intervals. Treatment with DAP-L and DAP-H did not significantly affect the body weight (*p* > 0.05) compared to the MC group body weight, indicating that the selected DAP doses did not cause apparent side effects in the mice (Figure 8A). Interestingly, the tumor growth in mice treated with DAP-L and DAP-H was significantly inhibited (*p* < 0.001) (Figure 8B). Additionally, photographs of the tumors showed that the tumor tissue morphology in the DAP-L and DAP-H groups was substantially different, and there was a significant reduction in tumor weight (*p* < 0.0001) compared to the control group (Figure 8C). The impact of DAPs on the morphology of lung cancer cells was shown via hematoxylin and eosin (H&E) staining (Figure 8D), with changes in the cell morphology, a noticeable decrease in the number of cell nuclei, and the appearance of areas of division and necrosis in the cytoplasm in the DAP-L and DAP-H groups. Moreover, the staining for Ki67, a marker linked to the recurrence and progression of malignant tumors, showed reduced expression in the tumor tissue following DAP treatment (Figure 8E). Yang et al. conducted an evaluation of the in vivo impacts of Rhein using the xenograft models and found that Rhein markedly decreased the viability of human NSCLC cells and stimulated apoptosis in a dose-dependent manner [35]. Li et al. found that Radix Tetrastigma flavonoid compounds could significantly downregulate Ki67 expression, indicating that Radix Tetrastigma flavonoids possess the capacity to restrict the proliferation of A549 cells [36].

### 3.9. Effect of DAP on Mice Organs

**Figure 9:**
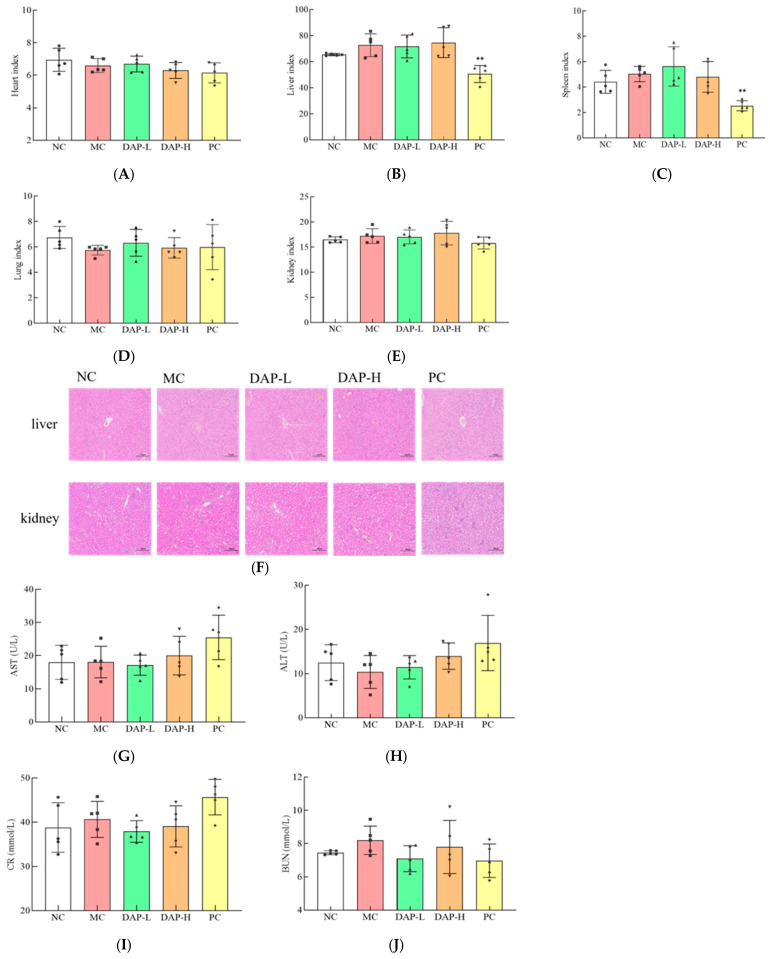
Effect of DAPs on mouse organs. (**A**) Hearts; (**B**) livers; (**C**) spleens; (**D**) lungs; (**E**) kidneys; (**F**) pathological evaluation of liver and kidney, scale bar: 200 μm; (**G**) AST; (**H**) ALT; (**I**) CR; (**J**) BUN; triangle, square, bullet represent the distribution of samples in each group; ** *p* < 0.01 compared to the MC group.

The hearts, livers, spleens, lungs, and kidneys of the mice were carefully excised. These organs were then weighed to determine the organ indices. Figure 9A–E illustrates that the organ indices for the heart, liver, spleen, lungs, and kidneys of mice treated with DAP-L and DAP-H did not differ significantly (*p* > 0.05) from those in the MC group, while the liver and spleen indices in the PC group were significantly decreased (*p* < 0.01). A histological examination of the liver and kidney tissues via HE staining revealed no pathological alterations in these tissues within any group compared to the MC group (Figure 9F). Furthermore, hepatic and renal function was assessed using blood biochemical assays. Figure 9G–J shows that no significant effects (*p* > 0.05) of DAP-L and DAP-H were detected on the levels of ALT, AST, BUN, and CR. All results suggest that DAPs could successfully inhibit the growth of lung cancer cells in vivo without having adverse effects on the mouse organs.

### 3.10. Effects of DAPs on Bax, Bcl-2, and Caspase-3 Proteins in the Tumor Tissue of Tumor-Bearing Mice

**Figure 10:**
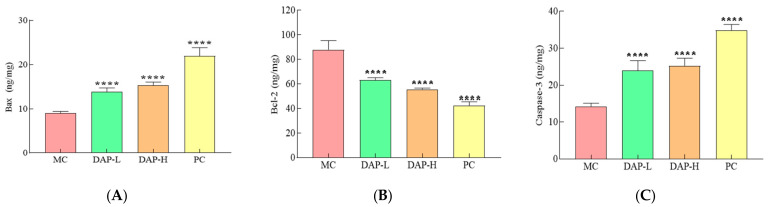
Expression of apoptosis-related proteins in tumor tissue. (**A**) Bax; (**B**) Bcl-2; (**C**) caspase-3. **** *p* < 0.0001 compared to the MC group.

To investigate whether cell apoptosis is connected to the anti-cancer action of DAPs in vivo, the amounts of the proteins Bax, Bcl-2, and caspase-3 were measured in the tumor tissues of mice with lung cancer. Figure 10A–C illustrates that following DAP-L and DAP-H treatment, there was a notable decrease in the Bcl-2 protein levels (*p* < 0.001) and a large increase in the Bax and caspase-3 levels (*p* < 0.001) within the tumor tissues. These results demonstrate that DAPs caused apoptosis in the A549 tumor cells in mice. Yi et al. proved that polyphenols derived from the Pinus koraiensis pinecone significantly promoted the expressions of Bax and activated caspase-3 and dramatically inhibited Bcl-2, leading to the activation of the mitochondrial apoptotic pathway in S180 tumor cells in mice [37]. Li et al. found that Radix Tetrastigma flavonoids could significantly downregulate the level of Bcl-2 and considerably upregulate the production of caspase-3, caspase-9, and Bax, suggesting that Radix Tetrastigma flavonoids could promote the apoptosis of A549 cells [36].

## 4. Conclusions

This research revealed that dried apricot polyphenols (DAPs) contain 949 phenolic substances, mainly including 2′,7-dihydroxy-3′,4′-dimethoxyisoflavan, scopoletin, rutin, quercetin-3-O-robinobioside, and elaidolinolenic acid. Dried apricot polyphenols have the potential capacity to inhibit the proliferation of A549 cells, reduce cell mobility, and arrest A549 cells at the G0/G1 and G2/M phases by lowering the expression levels of cyclin D1, CDK4, CDK6, cyclin B1, and CDK1. At the same time, dried apricot polyphenols could raise the intracellular ROS level to reduce the anti-apoptotic Bcl-2 levels and increase the levels of pro-apoptotic Bax and the apoptotic regulatory factors caspase-3 and caspase-9. This activates the mitochondrial intrinsic pathway and the cleavage of PARP1 protein, triggering apoptosis in the A549 cells. Moreover, dried apricot polyphenols could suppress the proliferation of tumors in tumor-bearing mice by reducing Ki67 expression, lowering the concentration of the anti-apoptotic protein Bcl-2 and elevating the levels of pro-apoptotic protein Bax and the apoptotic regulator caspase-3 in tumor tissues. Dried apricot polyphenols may serve as a possible resource in the fight against lung cancer.

## Data Availability

The original contributions presented in the study are included in the article/Supplementary Material, further inquiries can be directed to the corresponding authors.

## Supplementary Materials

The following supporting information can be downloaded at: https://www.mdpi.com/article/10.3390/foods14010108/s1, Table S1: Mass spectrometry detection parameters; Table S2: Analysis of dried apricot polyphenols; Figure S1. Total ion chromatogram (TIC) of dried apricot polyphenols (a: positive ion mode; b: negative ion mode).

## Abbreviations

DAPs	Dried apricot polyphenols
ROS	Reactive oxygen species
UPLC-MS/MS	Ultra-performance liquid chromatography-tandem mass spectrometry
NAC	N-Acetyl-cysteine
Bax	Bcl-2 associated X protein
Bcl-2	B-cell lymphoma-2
CDK	Cyclin-dependent kinases
Caspase	Cysteinyl aspartate specific proteinase
PARP1	Poly ADP-ribose polymerase 1
ELISA	Enzyme-linked immunosorbent assay
FITC	Fluorescein isothiocyanate
PI	Propidiumiodide
NC	Normal control group
MC	Model control group
PC	Positive control group
DAP-L	Low-dose DAP-treated group
DAP-H	High-dose DAP-treated group
ALT/GPT	Alanine aminotransferase
AST/GOT	Aspartate aminotransferase
CRE	Creatinine
BUN	Blood urea nitrogen
HE	Hematoxylin–eosin
