# Supplementary material for: Dried Apricot Polyphenols Suppress the Growth of A549 Human Lung Adenocarcinoma Cells by Inducing Apoptosis via a Mitochondrial-Dependent Pathway
Source: Foods. 2025 Jan 2;14(1):108. doi: 10.3390/foods14010108 (PMC11719503; doi:10.3390/foods14010108)

Supplementary Material

Table S1 Mass spectrometry detection parameters

Number	Compound	Molecular Formula	Ionization model	Retention time(min)	Collision energy(eV)	Declustering potential(eV)
1	2',7-Dihydroxy-3',4'-dimethoxyisoflavan	$C_{17}H_{18}O_5$	[M+H]+	2.9	30	50
2	Scopoletin	$C_{10}H_8O_4$	[M-H]-	4.1	-30	-50
3	Rutin	$C_{27}H_{30}O_{16}$	[M-H]-	3.7	-40	-40
4	Quercetin-3-O-robinobioside	$C_{27}H_{30}O_{16}$	[M-H]-	3.6	-40	-60
5	Elaidolinolenic acid	$C_{18}H_{30}O_2$	[M+H]+	8.8	30	50
6	Hamiltone A	$C_{18}H_{18}O_6$	[M-H]-	3.3	-30	-50
7	5-Glucosyloxy-2-Hydroxybenzoic acid methyl ester	$C_{14}H_{18}O_9$	[M-H]-	3.3	-30	-50
8	2,3,5,4'-Tetrahydroxystilbene-2-O-glucoside	$C_{20}H_{22}O_9$	[M-H]-	3.9	-30	-50
9	1-O-Vanilloyl-D-Glucose	$C_{14}H_{18}O_9$	[M-H]-	3.2	-30	-50
10	Delphinidin-3-O-(6''-O-p-coumaroyl)glucoside	$C_{30}H_{27}O_{14+}$	[M]+	3.8	30	50
11	Quercetin-7-O-rutinoside*	$C_{27}H_{30}O_{16}$	[M+H]+	3.7	30	50
12	Isoscopoletin	$C_{10}H_8O_4$	[M+H]+	4.1	30	50

Continue to table S1 Mass spectrometry detection parameters

13	Quercetin-3-O-galactoside	$C_{21}H_{20}O_{12}$	[M-H]-	3.6	-40	-60
14	6-Hydroxy-7-methoxycoumarin	$C_{10}H_8O_4$	[M+H]+	4.1	30	50
15	2,4,6,4'-Tetrahydroxy-stilbene-2-O-glucoside*	$C_{20}H_{22}O_9$	[M-H]-	3.6	-30	-50
16	Phthalic anhydride	$C_8H_4O_3$	[M+H]+	9.7	30	50
17	Quercetin-3-O-glucoside-7-O-rhamnoside*	$C_{27}H_{30}O_{16}$	[M+H]+	3.6	30	50
18	Piceatannol-3'-O-glucoside*	$C_{20}H_{22}O_9$	[M-H]-	3.5	-30	-50
19	Quercetin-3-O-neohesperidoside*	$C_{27}H_{30}O_{16}$	[M+H]+	3.5	30	50
20	Rutin Trihydrate	$C_{27}H_{36}O_{19}$	[M-3H_2O+H]+	3.9	30	50
21	Quercetin-3-O-(4''-O-glucosyl)rhamnoside*	$C_{27}H_{30}O_{16}$	[M+H]+	3.9	30	50
22	[6]-Gingerol	$C_{17}H_{26}O_4$	[M+H]+	7	30	50
23	Scopoletin-7-O-glucoside	$C_{16}H_{18}O_9$	[M+H]+	3	20	20
24	Diisobutyl phthalate*	$C_{16}H_{22}O_4$	[M+H]+	9.6	30	50
25	Hesperetin-5-O-glucoside	$C_{22}H_{24}O_{11}$	[M-H]-	3.8	-30	-60
26	6-Hydroxyluteolin 5-glucoside*	$C_{21}H_{20}O_{12}$	[M-H]-	3.6	-30	-50
27	Senkyunolide C	$C_{12}H_{12}O_3$	[M+H]+	9.5	30	50

Continue to table S1 Mass spectrometry detection parameters

28	Phellodenol H	$C_{17}H_{20}O_9$	[M-H]-	2.9	-30	-50
29	Hesperetin-3'-O-glucoside*	$C_{22}H_{24}O_{11}$	[M+H]+	4.1	30	50
30	Butyl isobutyl phthalate*	$C_{16}H_{22}O_4$	[M+H]+	9.6	30	50

Table S2 Analysis of dried apricot polyphenols

Number	Compound	CAS	Class I	Class II	Proportion(%)
1	2',7-Dihydroxy-3',4'-dimethoxyisoflavan	52250-35-8	Flavonoids	Other Flavonoids	2.5462±0.4771
2	Scopoletin	92-61-5	Lignans and Coumarins	Coumarins	2.2214±0.1403
3	Rutin	153-18-4	Flavonoids	Flavonols	2.0964±0.1070
4	Quercetin-3-O-robinobioside	52525-35-6	Flavonoids	Flavonols	2.0376±0.1708
5	Elaidolinolenic acid	28290-79-1	Phenolic acids	Phenolic acids	1.9966±0.0527
6	Hamiltone A	6626-61-5	Flavonoids	Flavanones	1.9047±0.0676
7	5-Glucosyloxy-2-Hydroxybenzoic acid methyl ester	-	Phenolic acids	Phenolic acids	1.8670±0.1049
8	2,3,5,4'-Tetrahydroxystilbene-2-O-glucoside	82373-94-2	Others	Stilbene	1.8342±0.1434
9	1-O-Vanilloyl-D-Glucose	-	Phenolic acids	Phenolic acids	1.7743±0.1130
10	Delphinidin-3-O-(6''-O-p-coumaroyl)glucoside	-	Flavonoids	Anthocyanidins	1.7010±0.1270
11	Quercetin-7-O-rutinoside*	147714-62-3	Flavonoids	Flavonols	1.6409±0.0874
12	Isoscopoletin	776-86-3	Lignans and Coumarins	Coumarins	1.6368±0.0415

Continue to table S2 Analysis of dried apricot polyphenols

13	Quercetin-3-O-galactoside	482-36-0	Flavonoids	Flavonols	1.6236±0.0412
14	6-Hydroxy-7-methoxycoumarin	-	Lignans and Coumarins	Coumarins	1.5939±0.0408
15	2,4,6,4'-Tetrahydroxy-stilbene-2-O-glucoside*	-	Others	Stilbene	1.5685±0.0635
16	Phthalic anhydride	85-44-9	Phenolic acids	Phenolic acids	1.5122±0.0849
17	Quercetin-3-O-glucoside-7-O-rhamnoside*	-	Flavonoids	Flavonols	1.4936±0.0409
18	Piceatannol-3'-O-glucoside*	94356-26-0	Others	Stilbene	1.4923±0.0541
19	Quercetin-3-O-neohesperidoside*	29662-79-1	Flavonoids	Flavonols	1.4613±0.0399
20	Rutin Trihydrate	207671-50-9	Flavonoids	Flavanols	1.4408±0.1288
21	Quercetin-3-O-(4''-O-glucosyl)rhamnoside*	59262-54-3	Flavonoids	Flavonols	1.4296±0.0838
22	[6]-Gingerol	23513-14-6	Phenolic acids	Phenolic acids	1.3613±0.0598
23	Scopoletin-7-O-glucoside	531-44-2	Lignans and Coumarins	Coumarins	1.3582±0.1426
24	Diisobutyl phthalate*	84-69-5	Phenolic acids	Phenolic acids	1.1827±0.0835
25	Hesperetin-5-O-glucoside	69651-80-5	Flavonoids	Flavanones	1.1565±0.0197
26	6-Hydroxyluteolin 5-glucoside*	-	Flavonoids	Flavones	1.1534±0.1152
27	Senkyunolide C	-	Phenolic acids	Phenolic acids	1.1486±0.0528

Continue to table S2 Analysis of dried apricot polyphenols

28	Phellodenol H	917092-48-9	Lignans and Coumarins	Coumarins	1.1204±0.0532
29	Hesperetin-3'-O-glucoside*	-	Flavonoids	Flavanones	1.1083±0.0663
30	Butyl isobutyl phthalate*	17851-53-5	Phenolic acids	Phenolic acids	1.1030±0.1504

Fig. S1 Total ion chromatogram (TIC) of dried apricot polyphenols (a: positive ion mode; b: negative ion mode)

a

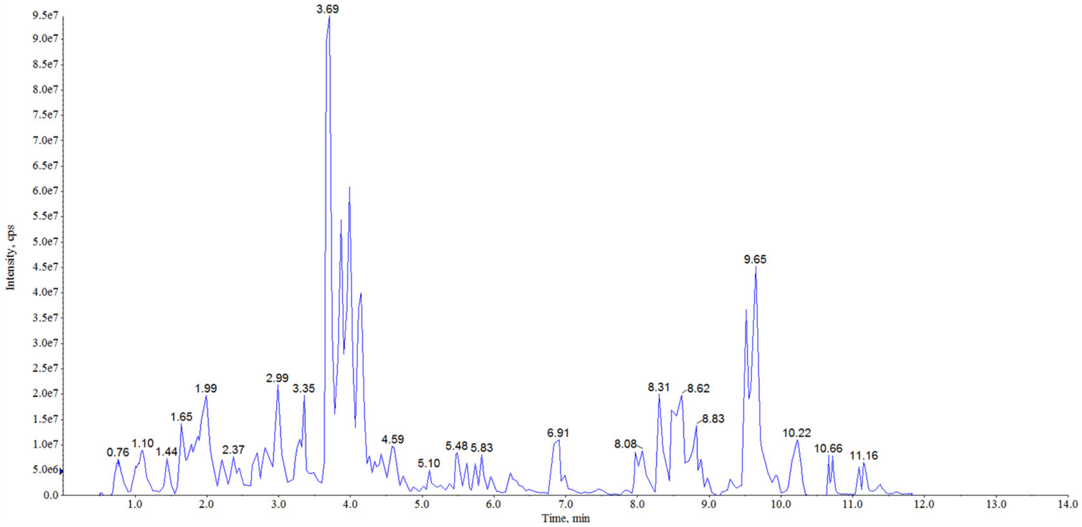

b

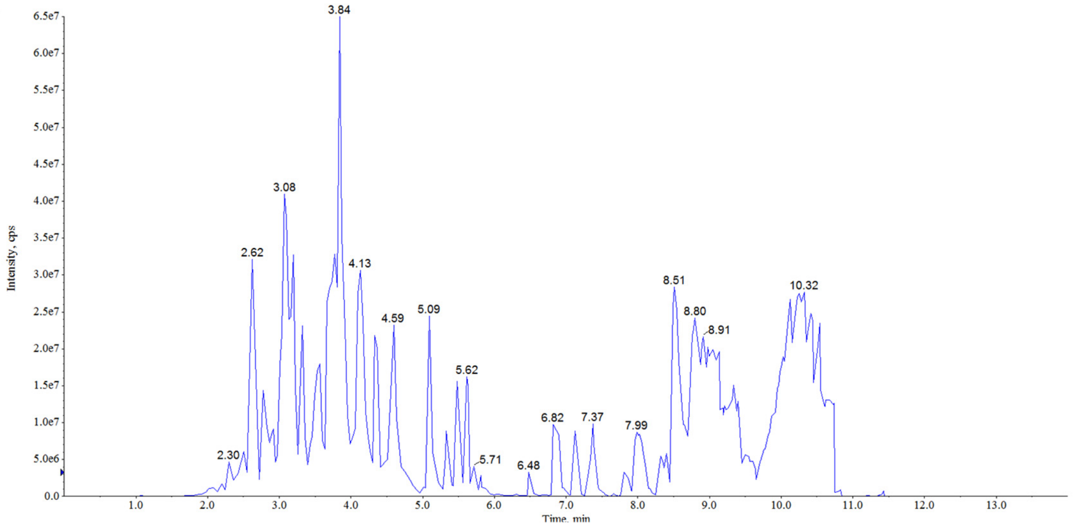

Supplement: Supplementary file 1 [file foods-14-00108-s001.zip › foods-3360465-supplementary.pdf]
